# Cellulose in Ionic Liquids and Alkaline Solutions: Advances in the Mechanisms of Biopolymer Dissolution and Regeneration

**DOI:** 10.3390/polym11121917

**Published:** 2019-11-21

**Authors:** Omar A. El Seoud, Marc Kostag, Kerstin Jedvert, Naved I. Malek

**Affiliations:** 1 Institute of Chemistry, University of São Paulo, 748 Professor Lineu Prestes Avenue, São Paulo 05508-000, SP, Brazil; marc_kostag@usp.br; 2 Bio-based Fibres, Materials and Production, Research Institutes of Sweden (RISE IVF), Box 104, SE-431 22 Mölndal, Sweden; kerstin.jedvert@ri.se; 3 Applied Chemistry Department, Sardar Vallabhbhai National Institute of Technology, Surat 395 007, Gujarat, India; navedmalek@yahoo.co.in

**Keywords:** cellulose solvents, mechanism of cellulose dissolution, solvatochromism, solvatochromic parameters, ionic liquids, quaternary ammonium electrolytes, salts of super-bases, mechanism of cellulose regeneration

## Abstract

This review is focused on assessment of solvents for cellulose dissolution and the mechanism of regeneration of the dissolved biopolymer. The solvents of interest are imidazole-based ionic liquids, quaternary ammonium electrolytes, salts of super-bases, and their binary mixtures with molecular solvents. We briefly discuss the mechanism of cellulose dissolution and address the strategies for assessing solvent efficiency, as inferred from its physico-chemical properties. In addition to the favorable effect of lower cellulose solution rheology, microscopic solvent/solution properties, including empirical polarity, Lewis acidity, Lewis basicity, and dipolarity/polarizability are determinants of cellulose dissolution. We discuss how these microscopic properties are calculated from the UV-Vis spectra of solvatochromic probes, and their use to explain the observed solvent efficiency order. We dwell briefly on use of other techniques, in particular NMR and theoretical calculations for the same purpose. Once dissolved, cellulose is either regenerated in different physical shapes, or derivatized under homogeneous conditions. We discuss the mechanism of, and the steps involved in cellulose regeneration, via formation of mini-sheets, association into “mini-crystals”, and convergence into larger crystalline and amorphous regions. We discuss the use of different techniques, including FTIR, X-ray diffraction, and theoretical calculations to probe the forces involved in cellulose regeneration.

## 1. Cellulose Dissolution and Regeneration: A Principal Strategy for Meeting the Increased Demand on Cellulosic Fibers

The demand for textile fibers is expected to rise at an annual rate of ca. 3.1% up to 2030 [1]. In 2018, 60% of the ca. 100 million tons of fibers employed worldwide were synthetic [2]. Due to their favorable properties, especially breathability and water absorbance (sweat), the demand on cotton and other man-made cellulosic fibers will continue to increase. Cotton, however, is a water-intensive and somewhat pesticide-intensive crop [3]. Therefore, its production will not match the increased demand, leading to the so-called “cellulose gap” [1]. Innovative strategies to produce cellulosic fibers from other sources, in particular wood cellulose, are being actively investigated; reuse and recycling of these fibers are becoming important issues [4].

**Figure 1:**
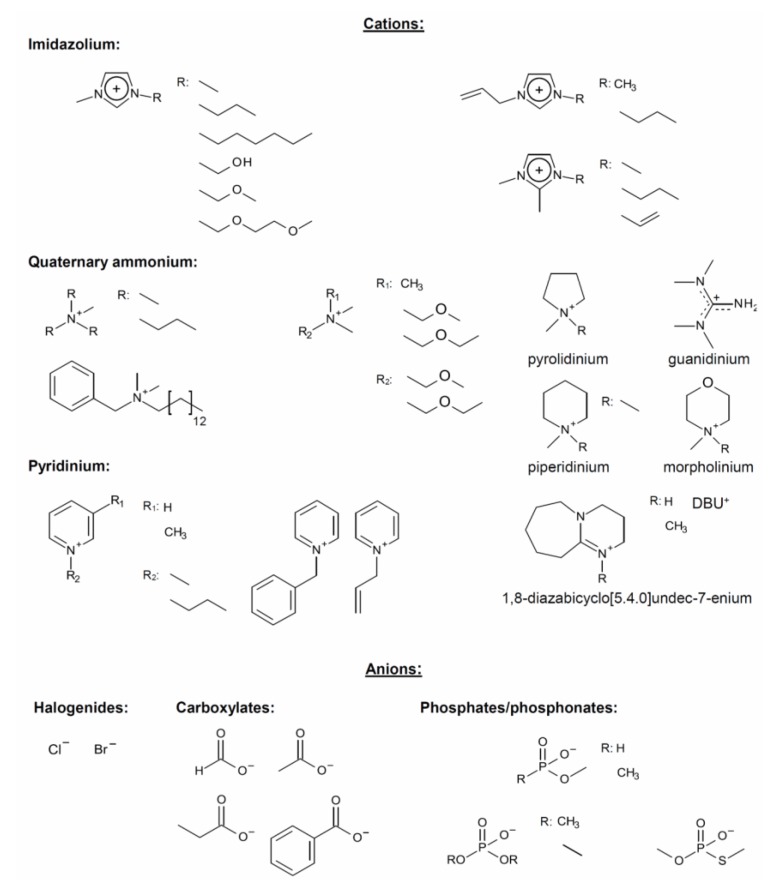
Molecular structures of anions and cations of typical ionic liquids employed as cellulose solvents and discussed in the present review, adapted from [5], with permission from MDPI, 2012.

This review covers some recent advances of the mechanisms of cellulose dissolution and regeneration from ionic liquids (ILs) in particular those based on imidazole, quaternary ammonium electrolytes (QAEs), salts of super-bases (Figure 1), and mixtures of these ionic compounds with molecular solvents (MSs). We will discuss the question regarding what makes a cellulose solvent an efficient one. We base our discussion on results from solvatochromism, NMR, and theoretical calculations. Additionally, we show how experimental data (FTIR, X-ray diffraction) and theoretical calculations are used to delineate the steps of cellulose regeneration.

## 2. Assessing Solvent Efficiency for Physical Dissolution of Cellulose

### 2.1. Ionic Liquids, Quaternary Ammonium Electrolytes, and Salts of Super-Bases: Versatile Cellulose Solvents

In addition to environmental and cost issues, a good solvent for cellulose should dissolve relatively high biopolymer concentrations, be chemically and thermally stable under the processing conditions, and can be recycled into the process by a scheme that is not energy intensive [6]. For efficient processing, the cellulose solution obtained should possess relatively low viscosity. Below, we focus our discussion of these issues on ionic liquids (ILs), and two of their sub-classes: quaternary ammonium electrolytes (QAEs), and salts of super-bases. Additional discussion on aspects of dissolution of cellulose and other natural polymers in ILs and applications of the solutions obtained therefrom can be found elsewhere [7,8,9,10,11,12,13,14,15,16,17].

**Figure 2:**
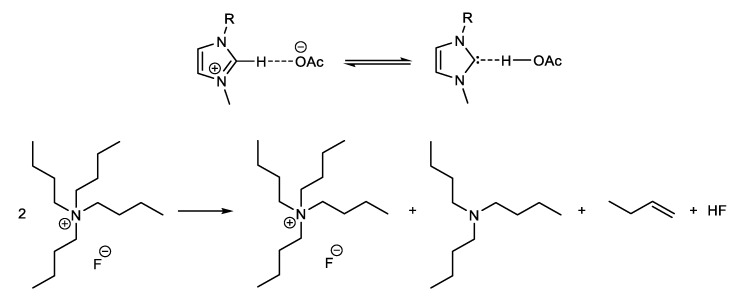
Formation of carbenes (**top**) and Hofmann elimination of quaternary ammonium compounds (**bottom**), redrawn from [21], with permission from Frontiers Media, 2018.

Depending on the structure of the anion and cation, some ILs and QAEs are more toxic, e.g., to aquatic life than chlorinated solvents; their biodegradation is relatively slow. These drawbacks can be attenuated/eliminated if the side-chains attached to the cation, and the anion carry hydrolysable functionalities, e.g., ester- and sulfate groups [18,19]. The cost issue of ILs versus molecular solvents (MSs) will decrease in relevance because of the expected increase of the production scale of the ionic solvents. Regarding thermal- and chemical stability, some of ILs commonly employed in cellulose chemistry, e.g., 1-(*n*-butyl)imizaolium X (BuMeImX; X = Cl^−^, PF_6_^−^, CH_3_SO_3_^−^) start thermal degradation in the 120–140 °C range, especially in the presence of water [20]. On the other hand, ILs and QAEs with basic anions (carboxylate, fluoride, hydroxide) may undergo thermal degradation and/or the proton elimination reactions shown below [21]. When formed, these carbenes react with the reducing end of cellulose, forming new carbon–carbon bond [22]. In fact, the absorption of acidic gases (CO_2_ and SO_2_) by ILs is based on the formation of carbene-gas adduct [23]. Hofmann elimination in QAEs, e.g., tetra(*n*-butyl)ammonium fluoride hydrate, TBAF·xH_2_O, leads to the formation of a trialkylamine [24]. The rates of these reactions increase as a function of increasing temperature; the liberated side products react with the substances present, e.g., (above-mentioned) carbenes with cellulose, and tertiary amines with acyl chloride or acid anhydride (see Figure 2). Therefore, the potential occurrence of thermal degradation and other side reactions should not be overlooked; the temperature employed for cellulose dissolution and subsequent derivatization in ILs and QAEs should be kept as low as feasible for the particular application.

A recent publication showed that cellulose thermal stability in BuMeImCl (24 h at 130 °C) is enhanced by co-solubilization of several amino acids, L-arginine being the most effective stabilizer. This was explained by formation of hydrogen-bonds (H-bonds) between the amino acid and cellulose that prevented the IL-cation, and its anion from forming three H-bonds simultaneously with three ipsilateral hydroxyls groups of the anhydroglucose units (AGUs). Formation of these “alternative” H-bonds successfully inhibit cellulose degradation [25]. This explanation should be reexamined because it is not likely that the 1-ethyl-3-methylimidazolium (EtMeIm) cation uses three hydrogens of different acidities (C_2_–***H***, C***H****_3_*, –C***H****_2_*–C_3_H_7_) [26], to bind efficiently to three hydroxyl groups in neighboring AGUs. Additionally, the possibility of thermal decomposition of the amino acids at 130 °C for 24 h should not be overlooked [27].

First generation ILs and QAEs are those synthesized by the reaction (nucleophilic substitution) of a tertiary amine, e.g., 1-metylimidazole and tri(*n*-butyl)amine with alkyl or benzyl halides. At room temperature, several of these compounds are either solids or liquids of high viscosity [28]. Note that the high viscosity of cellulose-IL solution adversely affects heat- and mass transfer; the problem is compounded if the solution shows Newtonian behavior, i.e., no shear-thinning is produced by increasing solution shear (agitation) [29]. Consequently, studying rheological properties such as viscoelasticity and flow behavior of cellulose solutions is important [30,31]. These properties are relevant because they allow process optimization, or reveal functional properties for biomedical applications e.g., tissue engineering [32]. As example, cellulose aerogel bead processing could be improved by using 1,5-Diazabicyclo[4.3.0]non-5-ene (DBN) propionate instead of 1-ethyl-3-methylimidazolium acetate (EtMeImAcO) as cellulose solvent because of higher intrinsic viscosity of the biopolymer solutions in the former solvent [33].

Two approaches were employed to attenuate this problem: “expansion” of the solvent volume by gas absorption (usually CO_2_); dilution of the IL with molecular solvents (MSs), usually dipolar aprotic ones that cause cellulose swelling, e.g., *N,N*-dimethylacetamide (DMAc), 1,3-dimethyl-2-imidazolidinone (DMI), and dimethyl sulfoxide (DMSO). Solvent expansion with CO_2_ requires that cellulose dissolution is carried out under pressure, albeit far below that required for supercritical conditions (73.9 bar at 31.1 °C) [34]. Dilution of the IL with a MS offers more flexibility because the physico-chemical properties of the binary solvent mixtures can be varied continuously by changing the relative concentrations of both components (IL and MS) [35]. Dilution with MSs leads not only to enhanced heat- and mass transfer, but also to better cellulose dissolution than in the pure IL or QAE, vide infra.

Recycling of the IL or QAE by a simple, low cost scheme is central to applications of these electrolytes in the chemical industry. In this regard, use of thermo-reversible ILs and phase separation are better alternatives to (energy intensive) evaporation of the volatiles present. Super-bases, in particular 1,8-Diazabicyclo[5.4.0]undec-7-ene (DBU), 1,5-Diazabicyclo[4.3.0]non-5-ene (DBN), and tetramethylguanidine (TMG) react with carboxylic acids (e.g., formic and acetic acids) forming salts that dissolve cellulose. These electrolytes are termed “distillable ILs” because the liquid super-base can be regenerated after the workout by heating to shift the equilibrium (super-base carboxylate ⇄ super-base + volatile carboxylic acid) to the right [36]. The same concept applies to the adducts between super-bases and CO_2_ [37]. Alternatively, separation of the IL from the medium was achieved by induced phase separation, liquid-liquid extraction, and membrane-based methods [38,39].

### 2.2. Solvatochromism and Calculation of Solvatochromic Parameters

The preceding discussion shows that the most important advantage of ILs as solvents for cellulose is their structural versatility, a consequence of the huge number of solvents that can be synthesized starting from the same “core” structure (e.g., the imidazole ring) by changing the attached side-chains and the corresponding anions. This flexibility means that the properties of the ionic solvents (miscibility with MSs, surface tension, viscosity, etc.) can “fine-tuned” according to necessity. The emphasis has now been shifted from establishing ILs as cellulose solvents to maximize their efficiency and reduce cost, so that they can be used on industrial scale. To address solvent efficiency, we discuss briefly the mechanism of physical dissolution of cellulose, and then show how the combined information from UV-Vis (solvatochromism), NMR, and theoretical calculations help in delineating the factors responsible for solvent performance.

**Figure 3:**
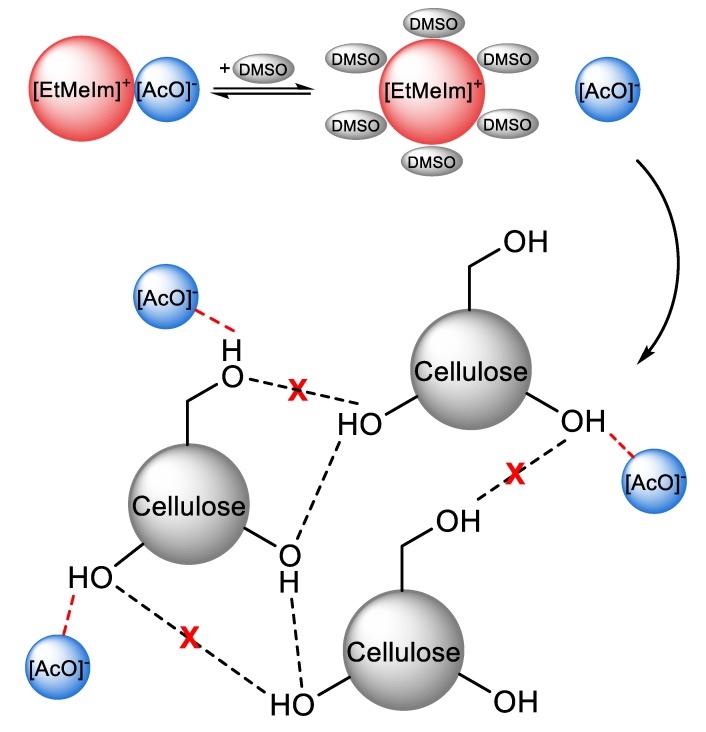
Schematic representation of the mechanism of cellulose dissolution in 1-ethyl-3-methylimidazolium acetate-dimethyl sulfoxide (EtMeImAcO-DMSO) mixture. The ionic liquid (IL) cation is solvated by DMSO and associated with the negatively charged cellulose-acetate ion H-bonded complex, redrawn from [41], with permission from Springer, 2016.

Physical dissolution of cellulose requires disruption of the inter- and intramolecular H-bonds between the hydroxyl groups of the AGUs, as well as the solvophobic interactions present [40]. Briefly, the dissolution proceeds by a cooperative mechanism: the interaction of the anion with the hydroxyl groups of the AGU leads to H-bond disruption and the development of a negative charge on cellulose. To maintain electric neutrality, the cations “condense” around the anion-cellulose complex. This leads to increased steric repulsion between the chains of the biopolymer-IL complex, and disruption of the solvophobic interactions between the AGUs, due to interactions of the latter with the (usually voluminous) cation. Most certainly, these IL-cellulose interactions proceed simultaneously rather than in the above-mentioned stepwise manner, as represented schematically in Figure 3 for the dissolution of cellulose in a mixture of EtMeImAcO-DMSO [41]. Thus, the charge density (hardness; Lewis basicity) and volume of the anion, the volume, rigidity, Lewis acidity, and hydrophobic character of the cation are determinant to cellulose dissolution [16].

Synthesizing a homologous series of ILs, QAEs, and salts of super-bases of varying molecular structures is not a demanding task. Examples are the synthesis of 1-R-3-methylimizaolium X where R = C_1_ to C_8_ and X is either the anion obtained by direct S_N_ substitution reaction (usually Br^−^ and Cl^−^) or after exchange for another anion, e.g., using macro-porous anion exchange resin. The efficiency of the synthesized series as cellulose solvents is then evaluated, and the dependence of the concentration of dissolved cellulose on the variable structural factor is assessed. We dwell here on some of the tools employed to explain the relative efficiency of these solvents.

At the outset, we discuss important practical aspects necessary for unambiguous rating of solvent efficiency. The purity of the IL and its dryness are important issues. In 71 ILs offered by a reagent supplier, the reported purities were (purity in %; number of ILs sold in %): 95, 21; 97, 28; 98, 25; only 7% of the ILs have purity ≥99%. The impurity present can be water, starting material (e.g., mono-substituted Im), or side products from the synthesis. These (mostly unspecified) impurities probably affect the reported wt% dissolved cellulose [7] and the rheological properties of the biopolymer-IL solution [42]. One approach is to increase the concentration of adventitious water to a certain, constant level, e.g., from 0.6 to 1 wt% [43]. This relatively small water concentration, however, inhibits the dissolution of cellulose in BuMeImCl [7]. Additionally, water interactions with ILs [44], and MSs are strong [45], and compete with the cellulose-solvent counterparts. This water-induced “leveling” effect may explain the little variation in the conductivity of BuMeImAcO on dilution with DMSO [43], in variance with the results of other studied, vide infra. Finally, addition of 1-methylimidazole to AlMeImAcO increased the wt% dissolved cellulose [46].

We consider the experimental protocol employed to assess cellulose dissolution, and the different scales of reporting dissolved biopolymer concentration. As there is no agreed upon protocol to assess cellulose dissolution, this experiment was done by manual-, magnetic-, and mechanical agitation, under (usually) unspecified conditions (stirring rate and time). Potential water uptake by the cellulose/IL solution during the experiment (e.g., that resulted from opening the vials to take samples for examination) is not commented upon. Likewise, biopolymer dissolution was judged visually, using a camera, a microscope, or by turbidity measurements [8,11,41,43,47]. Additionally, dissolution efficiency was reported using different composition scales, including, wt or volume percentage (100 × (wt_Cellulose_/[wt_IL_ + wt_MS_]) or 100 × (wt_Cellulose_/[V_IL_ + V_MS_]), and mole fraction χ. Recently, we introduced a specially constructed agitation system that ensures strong, sustained suspension agitation. We suggested a dissolution protocol based on visual inspection of the solution/suspension without opening the glass vial (LED light with a magnifying glass) and a microscope to reach final decision on cellulose dissolution. We showed that χ_AGU_ is an unambiguous scale to report solution composition, hence solvent efficiency [48]. For clarity, we stress that χ_AGU_ should refers to all components (cellulose + IL + MS) and not to the binary solvent mixture (IL + MS).

#### 2.2.1. Use of Solvatochromic Parameters to Assess the Efficiency of Cellulose Solvents

**Figure 4:**
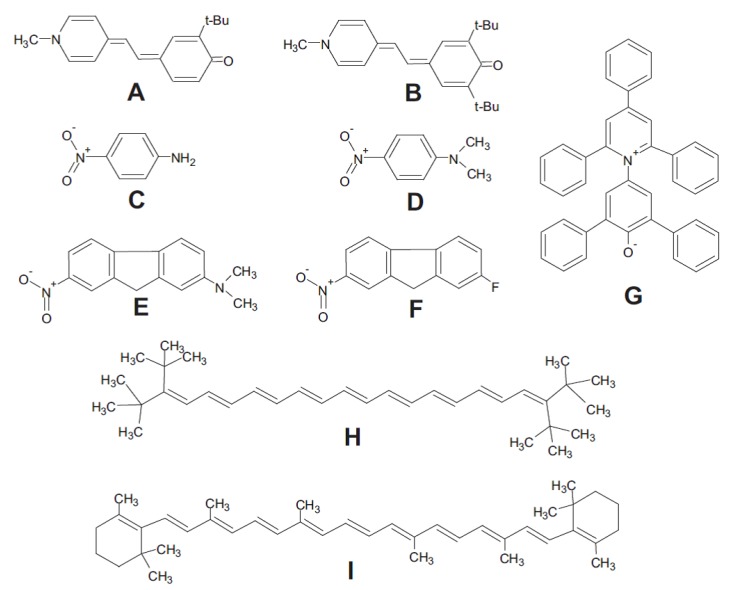
Typical examples of the probes that are employed for the determination of solvent descriptors. These include the pairs of homomorphs: *o*-*tert*-butylstilbazolium betaine (**A**) and *o*,*o*′-di-*tert*-butylstilbazolium betaine (**B**) (SA); 4-nitroaniline (**C**) and *N*,*N*-dimethyl-4-nitroaniline (**D**) (SB); 2-(*N*,*N*-dimethylamino)- 7-nitrofluorene (**E**) and 2-fluoro-7-nitrofluorene (**F**) (dipolarity/polarizability; D/P). The probes 2,6-diphenyl-4-(2,4,6-triphenylpyridinium-1-yl) phenolate (RB) (**G**); 3,20-di-*tert*-butyl-2,2,21,21-tetramethyl- 3,5,7,9,11,13,15,17,19-docosanonaene, ttbP9 (**H**); and (all *trans*) 1,1′-(3,7,12,16-tetramethyl-1,3,5,7,9,11,13,15,17-octadecanonaene-1,18-diyl)bis[2,6,6-trimethylcyclohexene], β-carotene (**I**) are employed for the determination of the empirical solvent (overall) polarity, and polarizability; reproduced from [49], with permission from Elsevier, 2013.

As discussed above, cellulose dissolution requires disruption of the H-bonds and solvophobic interactions present. Therefore, the ionic solvents and their mixtures with MSs should be relatively dipolar with good Lewis acidity (SA), Lewis basicity (SB) characteristics. These parameters can be quantified using solvatochromism. This term is used to denote the effects of solvents (pure and mixtures) on the UV-Vis and fluorescence spectra, hence colors, of certain compounds (probes) that are particularly sensitive to a special solvent property. Figure 4 shows some of the probes employed to quantify solvent properties relevant to cellulose dissolution.

**Figure 5:**
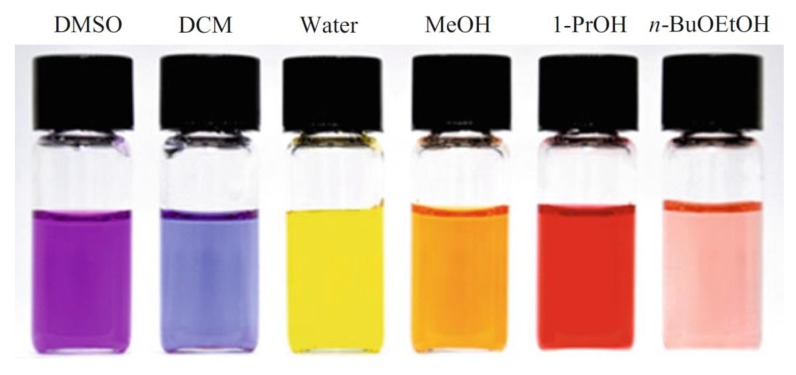
Colors of the solvatochromic polarity indicator 2,6-Dibromo-4-(E)-2-1(1- methylpyridinium-4-yl)ethenyl]phenolate in different solvents; DCM refers to dichloromethane; reproduced from [17], with permission from Springer,2018.

The use of these probes is based on the following principle discussed, for simplicity, for the zwitterionic RB. This probe has an intramolecular charge-transfer (CT, phenolate oxygen ⟶ quaternary ammonium nitrogen) that is stabilized in the ground state by the solvent. Upon absorption of light of the appropriate wavelength (λ) and subsequent excitation, the probe becomes a di-radical. The excitation time (femtosecond range) is much shorter than time required for the solvent molecules to rearrange (picosecond range). Consequently, the probe ground state CT is solvent-stabilized. The energy of this stabilization, hence Δ*E* (= *E*_Excited state_ − *E*_Ground state_) depends on the strength of probe–solvent interactions. Typically, the probe solutions in different solvents show distinct, vivid colors, e.g., those shown in Figure 5.

Values of *λ*_max_ for the intramolecular CT are then manipulated to calculate a parameter that describes the solvent property of interest, see e.g., Equation (1) for the empirical solvent polarity parameter:

*E_T_*(probe)/(kcal mol^−1^) = 28590.5/*λ*_max_ (nm), (1)

where *λ*_max_ is wavelength of the solvent-sensitive charge transfer band. The other parameters are calculated by a similar approach. Note that *E_T_*(probe) is the sum of a series of specific solvent properties, Equation (2).

*E_T_*(probe) = *a* SA + *b* SB + *d* SD + *p* SP, (2)

where S, SD, and SP refer to solvent, its dipolarity and polarizability, respectively and (*a, b, d, p*) are the corresponding regression coefficients. The signs of the latter coefficients indicate whether the solvent property in question favors (positive sign) or disfavors (negative sign) the particular probe–solvent interaction. On the other hand, the value of |regression coefficient| reflects the strength of the probe–solvent interaction.

In the Taft–Kamlet–Abboud approach, similar solvatotochromic parameters and different symbols were used to describe the probe–solvent interactions, *α*, *β*, and π* for solvent Lewis acidity, Lewis basicity, and (combined) dipolarity/polarizability. For consistency, we will use *SA*, *SB*, and *SD*/*SP* to describe the Taft–Kamlet–Abboud parameters, along the regression coefficients (*a, b*, and *d/p*) respectively (see Equation (3)):

*E_T_*(probe) = *a* SA + *b* SB + *d*/*p* SD/SP. (3)

Note that solvatochromic parameters were determined by a myriad of probes of different molecular structures. It is therefore not straightforward to compare probe response to solvent properties obtained by probes from different chemical classes; observing the trend is a more realistic approach [50,51,52].

#### 2.2.2. Pure Ionic Liquids as Cellulose Solvents

In many cases, cellulose dissolution in a series of ILs of related molecular structures is investigated. Either the cation is kept constant while the anion structure is varied, or vice versa. The dependence of wt% dissolved cellulose on the experimental variable (cation or anion) is then assessed using the above-mentioned solvatochromic parameters. Note that this assessment is limited to the structural variable studied, as shown by the following discussion on the relative importance of SA and SB. Published results showed that the relative importance of solvent descriptors is SB > SA for a series of BuMeImX, where X = Br^−^, Cl^−^, I^−^, AcO, F_3_CCO_2_^−^, NO_2_^−^, NO_3_^−^, CH_3_SO_3_^−^, CH_3_O-SO_3_^−^, C_8_H_17_O– SO_3_^−^, (CN)_2_N^−^, and SCN^−^ [53]. The same trend was observed for ILs that carry other heterocyclic rings, e.g., morpholinium and piperidinium. On the other hand, for ILs whose anion is weakly basic, e.g., bistriflimde (TF_2_N) the relative importance of solvent descriptors is SA > SB, independent of cation structure (imidazolium, piperidinium, morpholinium, etc.) [54,55]. Therefore, the dependence of cellulose dissolution in ILs on the solvatochromic parameters of Equation (2) (or Equation (3)) is related to which ion is being varied. This conclusion is in line with the high (Gutmann) acceptor numbers (related to SA) and donor numbers (related to SB) of ILs [56]; with cellulose dissolution in ILs [57], and results of theoretical calculations [58].

We now discuss some representative examples of cellulose dissolution in pure ILs. The efficiency of 1-ethyl-3-methylimidazolium electrolytes (EtMeImX; X = methyl phosphonate, methyl methyl phosphonate, and methylphosphate) in dissolving microcrystalline cellulose (MCC) was attributed partially to their relatively low SA (0.51 ± 0.01), high SB (1.0 ± 0.03), and SD/SP (1.05 ± 0.01). Thus high solvent basicity and dipolarity/polarizability are important for cellulose dissolution [59]. The same conclusion was extended to BuMeImX (X = formate, acetate, propionate, butyrate). These ILs are basic SB (1.1 ± 0.1) and dipolar SD/SP (0.98 ± 0.05), and not highly acidic SA (0.56 ± 0.01). Interestingly, although BuMeIm pivalate (X = (CH_3_)_3_CO_2_^−^) was found to be the most basic solvent (SA = 0.54; SB = 1.19), it dissolves less cellulose than the above-mentioned carboxylates, probably because of steric hindrance [60]. The efficiency of polysaccharide extraction from bran (the hard outer layers of cereal grain) by a series of 1-R-3-methylimidazolium methylphosphonate (R = ethyl, allyl, *n*-propyl, *n*-butyl) was attributed to a combination of their basicity (SB = 1.00 ± 0.01), dipolarity/polarizability (SD/SP = 1.03 ± 0.03), and low viscosity. The less viscous IL (R = Et; *η* = 107 cP) extracts 29 wt% polysaccharides after 2 h treatment at 50 °C, compared with 16% for the IL with R = *n*-butyl (*η* = 287 cP) under comparable conditions. The less viscus phosphinate IL (R = Et; *η* = 65 cP) extracts more polysaccharides than the corresponding methylphophonate [61]. The observation that cellulose is more soluble in mixtures of IL than in a single IL is worth mentioning. Examples are the dissolution of MCC in EtMeImCl + EtMeImAcO (at molar ratio 30:70), and in the eutectic mixture of EtMeImCl + BuMeImCl (at molar ratio 51:49 molar) at 50, 75, and 100 °C; more MCC dissolution was observed when DMSO was added to these IL mixtures [62].

#### 2.2.3. Binary Mixtures of Ionic Liquids-Molecular Solvents as Cellulose Solvents

The use of solvatochromic probes to rationalize cellulose dissolution was extended to binary mixtures of electrolytes with MSs. As dipolar aprotic solvents carry no acidic hydrogens [63], the above-mentioned binary mixtures have higher SB and SD/SP than SA [64]. Cellulose samples with different degree of polymerization (DP = 135, 288, 534, 582) were dissolved in binary mixtures of *N,N,N*-triethyl-*N*-octylammonium chloride (N_2228_Cl) in 22 MSs with very small SA, including strongly dipolar aprotic ones, e.g., DMAc, DMF, DMSO, acetonitrile, Sulf, and weakly dipolar ones, e.g., ethyl acetate, tetrahydropyran, *N*-methylpyrrol, 1-pentatnone. Cellulose was found to be soluble in QAE/MS with SB values >0.5 [65]. Cellulose was dissolved in mixtures of BuMeImAcO and EtMeImAcO with MSs, including DMAc, DMSO, DMI, tetramethyl urea, and Sulf. The emphasis was to probe the dependence of wt% dissolved cellulose on molar fraction of the IL *χ*_IL_ at 100 °C. As shown by the values of *E_T_*(RB), both ILs are more polar than the MSs studied. The dependence of SA, SB, and SD/SP on *χ***_IL_** was examined. As a function of increasing the latter, SA, SB, and SD/SP increase first relatively fast, then much slower close to *χ*_IL_ ca. 0.3, at which fast dissolution of MCC was observed (10 wt% MCC in a few minutes; 100 °C) [64]. The relative importance of *E_T_*(RB), SA and SB to dissolution of MCC and kraft pulp by ILs, and the effect of added water on cellulose solubility were investigated. The ILs employed included EtMeImX (X = (MeO)MePO_2_, (MeO)_2_PO_2_, NO_3_^−^, ClO_4_^−^, Tf_2_N^−^, PF_6_^−^, MeOSO_3_^−^), TMGH^+^X^−^ (X = AcO^−^, PrO^−^), BuMeImX (Cl^−^, AcO^−^, PrO^−^, butanoate, BuO^−^, (MeO)_2_PO_2_^−^) and *N*-methylmorpholine-*N*-oxide, NMMO. In most cases, 1 wt% dissolved cellulose was precipitated (regenerated) with ca. 1–3 moles of water/mole of IL. Added water increased the values of *E_T_*(RB) and SA fast until molar ratio water/IL ca. 0.25, then much slower as more water is dissolved. The same cellulose non-solvent increased SD/SP continuously, but decreased SB continuously. The solvents that dissolve cellulose efficiently are those associated with high “net SB” (SB-SA >0.4) [57]. The same research group extended their studied to include distillable ILs (recovery at 1 mbar, 170 °C), based on the carboxylate salts of heterocyclic bases (e.g., pyridine and 4-*N,N*-dimethylaminopyridine) and super-bases (including DBN, DBU, and TMG). The term super-base is defined as “neutral organic base, more basic than NaOH”. EtMeIm caboxylates were also considered salts of super-bases because of the ease of C_2_-***H*** proton abstraction to form isolable carbene. The solvent was considered efficient if it dissolves 5 wt% pre-hydrolyzed kraft pulp at 100 °C. All carboxylate salts of the super-bases (SB-SA = 0.46 to 0.59) dissolved cellulose, all salts of ordinary bases (SB-SA not available) did not pass this (qualitative) test. It was shown that H-bond donation and acceptance by the IL are relevant to cellulose dissolution [47].

The formation of carbenes from ILs with basic anions was demonstrated with an experiment where CO_2_ was used as non-solvent for cellulose precipitation from binary mixtures of BuMeImAcO with MSs (DMF, DMI, DMSO). Comparison of the ^1^H, ^13^C and ^15^N NMR spectra of the starting cellulose/IL-MS solution with those after contact with CO_2_ (up to 22 MPa) showed changes in the chemical shifts (δs) of the above-mentioned nuclei. These changes were explained on the bases of addition of the gas to the formed carbene to produce BuMeIm-CO_2_-AcOH (IL-CO_2_ adduct associated with AcOH). Formation of this species resulted in a decrease in SB and SD/SP and an increase in SA, hence a decrease in (SB-SA), leading to cellulose precipitation. This simple strategy of cellulose regeneration may be more environmentally attractive than the addition of an non-solvent [37].

**Figure 6:**
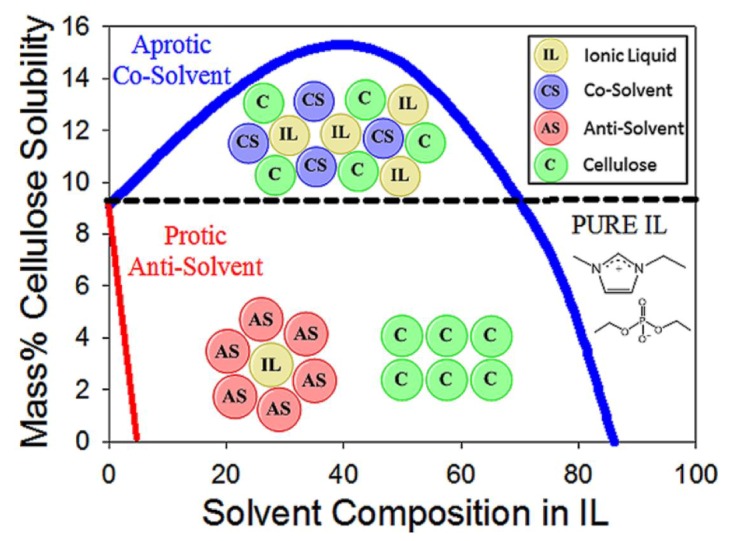
Preferential solvation of the IL ions by co-solvents and non-solvents. Preferential solvation of the IL ions with the non-solvent drives it out of the solvation layer of the biopolymers, leading to its precipitation, reproduced from [66], with permission from ACS,2016.

The effects of co-solvents (DMF, DMI, DMSO) and non-solvents (water, MeOH, EtOH) on the dissolution of MCC in BuMeImCl and EtMeIm diethylphosphate (EtMeIm(EtO)_2_PO) were investigated in the range 40–120 °C. Mixtures of ILs-MS dissolved more MCC than the pure ILs. Addition of non-solvents caused cellulose precipitation in the order water > MeOH > EtOH. The effects of both classes of solvents on the solvatochromic parameters were determined as a function of binary mixture composition (in m%). Whereas SB changed only slightly as a function of increasing wt% of the co-solvent, it decreased fast as a function of increasing m% of the non-solvent. The corresponding SA values showed an inverse trend, sharp increase as a function of increasing wt% of the non-solvent, more gradual increase as a function of increasing the wt% of the co-solvents. Therefore, addition of the former solvents leads to a sharp decrease of (SB-SA). This is due to preferential solvation of the IL ions, as indicated by the decrease of IR υ_P = O_ band, and downfield shift of the ^1^H δ of the C_2_-***H*** of the imidazolium ring of EtMeIm(EtO)_2_PO. Both spectroscopic data explain cellulose precipitation that accompanies the addition of non-solvents. The effects of both classes of solvents are depicted in Figure 6 [66]. Molecular dynamic (MD) calculations on cellulose dissolved in pure BuMeImAcO and aqueous IL indicated that the water molecules “intercalate” between the anion and the hydroxyl groups of the AGU. This drives both ions of the IL outside the first solvation layer of cellulose, leading to its precipitation [67].

The dissolution of MCC, cotton, wood pulp- and bamboo pulp cellulose in mixtures of EtMeOmAcO and the green MS propylene carbonate (PC) was studied at 50 and 80 °C. At 80 °C these cellulose samples dissolved at *χ*_IL_ = 0.3, and rapidly at *χ*_IL_ = 0.4. At 50 °C; 4–8 wt% cotton cellulose dissolved at *χ*_IL_ = 0.5. Although values of *E_T_*(30), SA, SB, SD/SP of the IL are larger than those of PC, the largest increase observed on dissolving the IL in PC was that of SB. These solvatochromic results show the importance of solvent basicity for cellulose dissolution [68].

**Figure 7:**
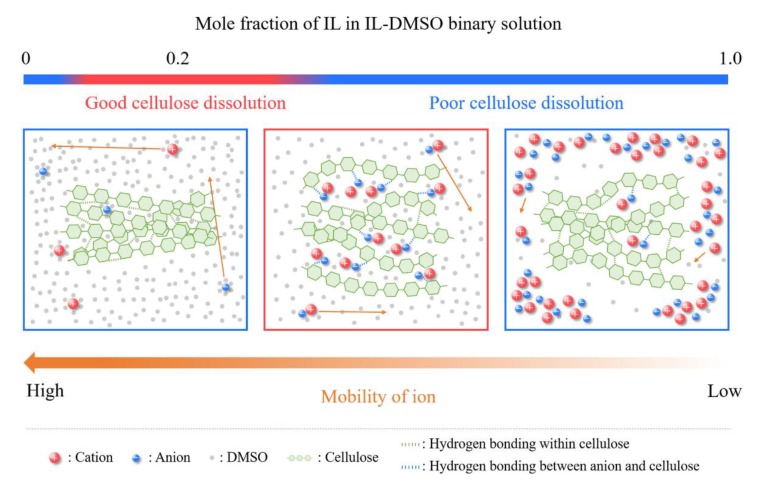
Schematic representation of the effect of binary mixture composition on cellulose dissolution. The left part is at high IL dilution with DMSO, the central part is at *χ*_IL_ ca. 0.2 where maximum microcrystalline cellulose (MCC) dissolution occurs. Further dilution with the molecular solvent (MS) solvent results in sharp decrease of cellulose solubility. Representation based on conductivity and solvatochromic data, reproduced form [71], with permission from Elsevier2019.

Published results showed that the dependence of cellulose dissolution in IL-MS mixtures on the concentration of the MS (reported using different scales, e.g., wt% and *χ*_IL_) is not linear, but shows a maximum at a certain solvent composition [62,69,70]. A rationale for this non-linear dependence is shown in the following schematic representation for the dissolution of MCC in EtMeImX-DMSO (X = AcO^−^; diethylphosphate, methylphosphonate; BF_4_^−^; Figure 7). At higher IL concentration (part on the right), the low mobility of ions restricts the IL from penetrating between the cellulose chains, resulting in relatively poor cellulose dissolving ability. Upon diluting the ionic liquid by DMSO, MCC dissolution is gradually enhanced due to the concomitant increase in ion mobility, while the SA value (ca. 0.8) remains constant (central part of Figure 7). However, the cellulose dissolving ability sharply decreases below *χ*_IL_ ca. 0.2, although the mobility of the ions is relatively high (left part of Figure 7). Probably the efficient solvation of the IL ions by DMSO competes with the “solvation” of the IL by cellulose [71].

Published data indicate a connection between cellulose dissolving efficiency of the solvent and effect of the latter on the reactivity of the dissolved biopolymer, e.g., in derivatization. Thus, the difference in the rate constants and activation parameters for the acetylation of MCC in mixtures of the IL 1-allyl-3-methylimidazolium chloride (AlMeImCl) with DMSO, DMAc, and Sulf was attributed to macroscopic- and microscopic properties. Solution viscosity is an important example of the former. Solutions of MCC in IL-DMSO are less viscous than in IL-Sulf. This affects the rates of diffusion of the species present (calculated by MD simulations), hence reactivity. On the microscopic level, MCC/IL-DMSO solutions possess higher empirical polarity *E_T_*(WB), and SB than MCC/IL-Sulf under comparable conditions [72]. Similar conclusions were reached for Im-catalyzed esterification of MCC. The acylating agents were *N*-acetyl- to *N*-hexanoylimidazole, obtained by reacting Im with carboxylic acid anhydrides in mixture of AlMeImCl with DMAc, DMSO, and Sulf. The observed order of rate constants was: IL-DMSO > Il-DMAc > IL-Sulf. The reactivity in IL-DMSO relative to IL-Sulf (solvents that carry the S=O dipole) was explained by a combination of lower viscosity, higher *E_T_*(WB), and SB of the MCC/IL-MS [73].

**Figure 8:**
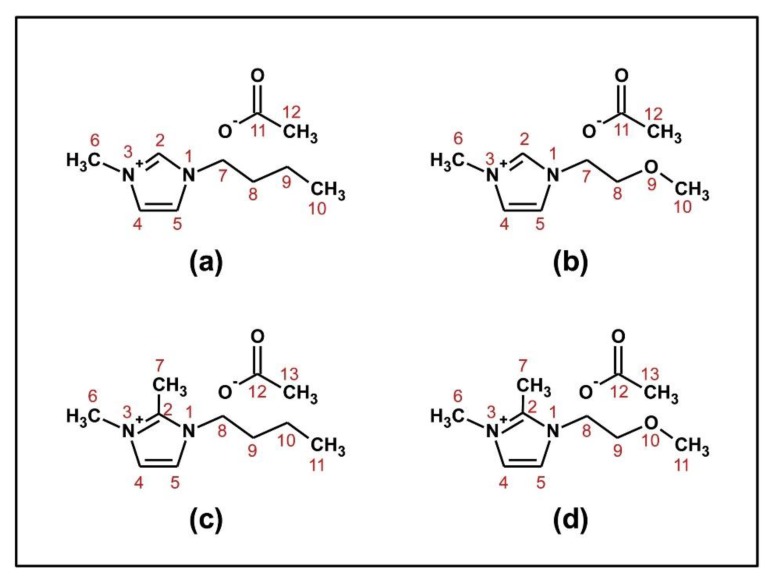
Molecular structures of IL without, and with ether oxygen in their side chains: (**a**) 1-butyl-3-methylimidazolium acetate, (**b**) 1-(2-methoxyethyl)-3-methylimidazolium acetate, (**c**) 1-butyl-2,3-dimethylimidazolium acetate, (**d**) 1-(2-methoxyethyl)-2,3-dimethylimidazolium acetate; reproduced from [74], with permission from Elsevier,2019.

In an attempt to increase efficiency, ILs with ether oxygen in their side-chains were employed as solvents for cellulose dissolution and acylation (see Figure 8). We expected that the IL 1-(2-methoxyethyl)-3-methylimidazolium acetate (PrOMeImAcO; compound b of Figure 8b) should be more efficient than BuMeImAcO (Figure 8a) because of the presence of an additional Lewis base in its structure (the ether oxygen). Surprisingly, pure BuMeImAcO and its binary mixtures with DMSO were more efficient cellulose solvents than their PrOMeImAcO counterparts. For example, at 60 °C and *χ*_DMSO_ = 0.6, the solubilities were 16, 6 wt% (BuMeImAcO-DMSO), and 13, 4.5 wt% (PrOMeImAcO-DMSO), for MCC and eucalyptus pulp, respectively. Microwave-assisted acylation by *N*-acteyl- and *N*-benzoylimidazole was carried out at 60 °C for 2 h, *χ*_DMSO_ = 0.6, *N*-acylimidazole/AGU = 1.5/1. Again, the DS values of the isolated esters were larger in case of BuMeImAcO-DMSO than PrOMeImAcO-DMSO (DS = 1.64 and 0.81, respectively). To explain this difference, we measured rheology and ^1^H, ^13^C NMR chemical shifts as a function of solution composition. Rheology measurements of MCC/IL-DMSO showed that the BuMeImAcO solutions are less viscous and possess lower energy of viscous flow than their PrOMeImAcO counterparts possess. Solvatochromic data showed that the order of (*SB*-*SA*) is BuMeImAcO-DMSO (ca. 0.86) > PrOMeImAcO-DMSO (ca. 0.75) over the composition range *χ*_DMSO_ = 0.3–0.7, i.e., the former binary mixture is more basic. Finally, the dependence of the IL NMR chemical shifts on the concentration of cellobiose (CB, a model for cellulose) showed larger slopes for C_2_–***H***, C***H_3_***CO_2_^−^ (^1^H), ***C_2_***–H and CH**_3_*C***O_2_^−^ (^13^C) for BuMeImAcO. That is, the latter IL forms stronger H–bonding with CB.

**Figure 9:**
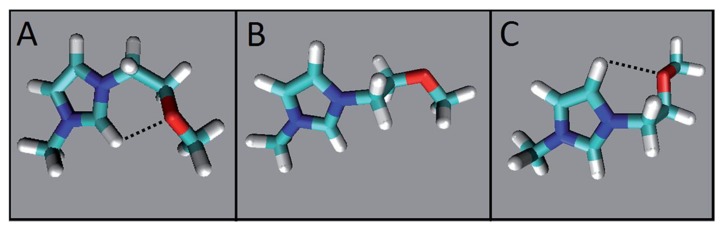
Molecular dynamics (MD) simulation-based snapshots of some limiting conformations of the cation of 1-(2-methoxyethyl)-3-methylimidazolium acetate in IL–water *χ*_w_ = 0.67. The colors used to denote the atoms are red, cyan, white, and navy blue for oxygen, carbon, hydrogen, and nitrogen, respectively. The distances between the atoms are (in nm) O···H–C_2_ = 0.239 (**A**); O···H–C_2_ = 0.468 ((**B**); open structure); O···H–C_5_– = 0.243 (**C**), reproduced from [75], with permission from RSC, 2017.

The lower efficiency of PrOMeImAcO was attributed to simultaneous “deactivation” of the ether oxygen (Lewis base) and C_2_-***H*** (Lewis acid) of the imidazolium ring due to intramolecular hydrogen bonding, as shown in Figure 9, based on MD calculations [75].

To probe this hypothesis (deactivation of the H-bonding between the ether linkage and C_2_–***H***), ILs with C_2_–***CH_3_*** instead of C_2_–***H*** were studied, namely, 1-butyl-2,3-dimethylimidazolium acetate (Figure 8c) and 1-(2-methoxyethyl)-2,3-dimethylimidazolium acetate (Figure 8d). Although this blocking did not change the values of (SB-SA) noticeably for *χ*_DMSO_ = 0.3–0.6, nor the order of (SB-SA), it led to enhanced cellulose dissolution for both IL-DMSO mixtures, without a noticeable effect on biopolymer reactivity in the acylation reaction. An interesting observation was that the acylation with *N*-benzoylimidazole in these IL-DMSO mixtures produced appreciable amounts of cellulose acetate. The DS_Acetate_/DS_Benzoate_ were 0.45 and 1.54, for BuMeImAcO-DMSO, and PrOMeImAcO-DMSO, respectively [74]. The only source of acetate is the IL, another manifestation that that ILs and their solutions in MSs are not always “spectators” [76].

The preceding representative examples show that solvatochromism can be fruitfully employed to provide some guidelines for choosing solvents for cellulose. These should form efficient hydrogen bonding to the hydroxyl groups of the AGUs, with the solvent or binary mixture acting as Lewis acid/base. Relatively high values of SD/SP, low viscosity, and higher temperatures favor cellulose dissolution and derivatization. Unfortunately, we were unable to assess the relative importance of solvophobic interactions from available solvatochromic data because there is no clear approach to split the SD/SP parameter into its components.

### 2.3. Use of NMR and Theoretical Calculations to Assess Ionic Liquids and Their Mixtures with Molecular Solvents as Cellulose Solvents

Other techniques, in particular NMR (chemical shifts, δs, longitudinal-, T_1_, and traverse, T_2_ relaxation time) were used to gain insight into cellulose–solvent interactions, hence, to corroborate the above-mentioned guidelines for efficient biopolymer solvents. The relatively high viscosities of cellulose solutions in ILs may lead to severe ^1^H line broadening. Consequently, CB is usually used as cellulose model in most of these studies. Likewise, working with viscous CB-IL solutions was avoided by diluting with deuterated solvents, in particular DMSO-*d*_6_. Unlike cellulose, CB is soluble in dipolar MSs. Hence, NMR data, e.g., δ, report on CB dissolved by DMSO and the IL-DMSO complex. If these solute–solvent interactions are independent, then values of (*δ*_CB/IL-DMSO_ − *δ*_CB-DMSO_) reflect CB–IL interactions. The implicit assumption is that these are also responsible for cellulose dissolution in IL-MS mixtures.

^13^C and ^35/37^Cl NMR T_1_ and T_2_ measurements were carried out on glucose, glucose pentaacetate (no free hydroxyl group), and CB dissolved in BuMeImCl. The results demonstrated that the solvation of the mono-and disaccharide by the IL involves H-bonding between the carbohydrate hydroxyl protons and the IL chloride ions in a 1:1 stoichiometry [77]. ^1^H and ^13^C NMR δs of CB dissolved in EtMeImAcO-DMSO-*d*_6_ were measured as a function of increasing the molar ratio IL/CB from 1 to 30. The data obtained clearly suggested the formation H-bonding between the hydroxyl groups of CB with the acetate anion and the relatively acidic C_2_–***H*** of the imidazolium ring. Additional evidence for this H-bonding was obtained from the data of CB octaacetate (no free hydroxyl groups). The latter is sparingly soluble in the IL, even at 100 °C. Unlike CB, values of ^1^H and ^13^C δs of this derivative were insensitive to increasing the concentration of the IL [78]. Dissolution of MCC in 17 ILs at 80 °C was studied. The anions were acetate (11 ILs), chloride (3 ILs), BF_4_^−^ and PF_6_^−^. The cations were 1-R-3-MeIm^+^; R = Et, Pr, Bu, Al, and C_6_-C_16_, 1-R-1,2-Me_2_Im^+^. MCC was soluble in the 1-R-3-MeIm^+^ series, the solubility decreased as a function of increasing the length of R. It was found to be insoluble in IL based on 1,2-dimethylimidazole and those with BF_4_^−^ and PF_6_^−^ anions. This relative order of solubility was explained based on ^1^H and ^13^C chemical shift data of the IL and the CB dissolved in IL-DMSO-*d*_6_. Data analysis indicated that H-bonding between the hydroxyl groups of the disaccharide and the anion/cation of the IL represent the major solute–solvent interactions, with the IL cation and anion acting as Lewis acid and Lewis base, respectively. When these H-bonding abilities were impaired, e.g., by increasing steric volume of the cation (R from Et ⟶ C_16_) or by decreasing the basicity of the anion (AcO^−^ ⟶ BF_4_^−^), the changes in δs decreased greatly, indicating little disaccharide–IL interactions. These conclusions about the effects of the molecular structures of the ILs on solute–solvent interactions were extended to MCC dissolution in the different ILs [79].

FTIR, 1D ^1^H and ^13^C, and 2D NMR spectra (^1^H–^13^C HSQC) were employed to investigate the absorption of CO_2_ by solutions of MCC dissolved in cold NaOH solution (8 wt%; −5 °C) without, or with previous treatment of cellulose with DBU. The solvent for the NMR experiment was EtMeImAcO-DMSO-*d*_6_. Cellulose was regenerated from these solutions by addition to water and ethanol. Only the cellulose regenerated from the latter solvent showed the presence of a carbonate ion peak (IR, 1593 cm^−1^); this CO_2_ chemisorption is reversible, i.e., physical. Cellulose regeneration in ethanol is presumably faster than in water. This faster/stronger biopolymer chain contraction is presumably the reason for ethanol being able to preserve the chemisorbed CO_2_. This gas chemisorption was observed for samples without, or with pretreatment with the super-base DBU; it should be considered when cellulose is treated with alkali solutions [80]. Finally, it is worth mentioning that NMR was used to probe the deleterious effect of adventitious water on cellulose solubility in ILs and IL-MSs. An example of this use is measurement of δ and peak width at half-height (= T_2_ × 1/π) of water protons and (^19^F) as a function of the water concentration in solutions of cellulose in TBAF/DMSO-*d*_6_. The biopolymer is soluble in this solvent because the Cel–OH···F^−^ bonds are stronger than cellulose-cellulose H-bonds. Water preferentially solvates the F^−^ ions at the expense of F^−^···HO–Cel interactions, this leads to reformation of the cellulose-cellulose H-bonds, and eventually to solution gelation and cellulose precipitation [81]. More information on the use of NMR to probe the mechanism of cellulose dissolution can be found in a recent review article [82].

In addition to corroborating the conclusions about cellulose dissolution in ILs and ILs-MSs, the results of theoretical calculations, in particular molecular dynamic simulations (MD) and density functional theory (DFT), furnish data that are experimentally laborious/difficult to obtain, or inaccessible. This includes, *inter alia*, optimized geometry in gas- and liquid phases, charges on atoms, average distances between the atoms of interest, e.g., Cel–O–H···anion, Cel–O(H)···cation, diffusion coefficients of the species of interest, and average numbers of H-bonds. For cellulose dissolution in IL-MSs, the results of MD also indicate the relative composition of the solvation layer (set at a certain, arbitrary distance from the surface of the cellulose ensemble). Additionally, plots of radial distribution function (rdf or g(r)) versus distance using atoms of both the cellulose ensemble, and the solvent system indicate the strength of the biopolymer–solvent interactions, an important information for rationalizing the relative efficiency, e.g., of two IL-MS systems.

We stress that validation is required for obtaining robust MD data, e.g., by comparing the theoretically calculated solution density with the experimentally determined one. Considering that the solutions of cellulose in IL-MS are relatively viscous (leading to slow diffusion of the species), and contain ions, it is recommended that an “annealing” procedure be employed to guarantee the robustness of the calculated results. This is done by performing cycles of “heating/cooling of the material” in the simulation box, followed by comparing certain MD-calculated properties, e.g., solution density or potential energy with those obtained in the first, or equilibration phase. Good agreement between these sets of parameters (before and after annealing) indicates reliable results [75,83].

**Figure 10:**
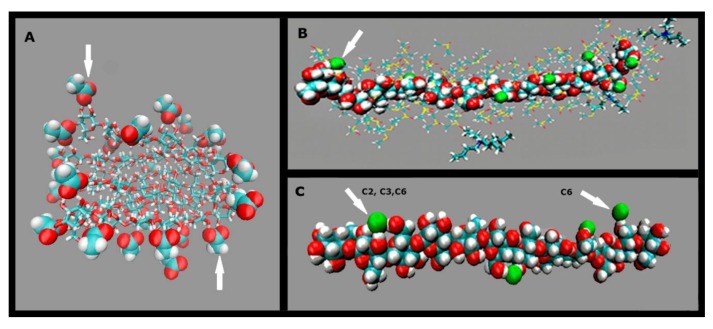
Molecular dynamic-based snapshots for the dissolution of cellulose oligomers by in IL-DMSO. Parts (**A**–**C**) are for the following ILs, respectively: diallylbenzylmethylammonium acetate (unpublished data), tetra(*n*-butyl)ammonium fluoride (TBAF), 1-allyl-3-methylimidazolium chloride (AlMeImCl); adapted from [73,84], with permission from Elsevier, 2014 and 2015.

An important result of MD that bears on the efficiency of ILs as cellulose solvents is that the anion can form H-bonds to more than one hydroxyl group of the same or neighboring AGU units, as shown by the following snapshots (Figure 10). Part (A) indicates the simultaneous H-bonding of the oxygen atoms (red) of the acetate anion of diallylbenzylmethylammonium acetate (in IL-DMSO) to Cell-OH (unpublished results). Parts (B) and (C) are for cellulose dissolution by TBAF·3H_2_O/DMSO [84] and AlMeImCl-DMSO [73]; in both cases the halide ion is represented in green color. As can be seen, the AcO^−^ anion from H-bonds to C_2_–OH and C_3_–OH of the same AGU (Figure 10A, upper arrow) or two hydroxyl groups of adjacent AGU (Figure 10A, lower arrow). Similar H-bonding is also observed for the F^−^ of TBAF (Figure 10B) and the Cl^−^ of AlMeImCl (Figure 10C). The efficiency of this H-bonding can be shown by considering the MD results for the dissolution of glucose dodecamer (oligomer)/R_4_NF-hydrate/DMSO. Thus, the extension of the oligomer solvation layer by (F^−^), 0.122 to 0.196 nm, is not too far from the distances between (F^−^) and water in crystals of hydrated inorganic electrolytes, calculated from X-ray diffraction (0.162–0.185 nm). On the other hand, the number of H-bonds of the oligomer OH groups and (F^−^) are 7, and 16, for dibenzyldimethylammonium fluoride·0.1H_2_O-DMSO, and TBAF·3H_2_O-DMSO, respectively [72]. This simultaneous, efficient H–boding is at the expense of the intra- and intermolecular H–bonding in cellulose, leading to its dissolution.

A similar picture emerged from MD results on the interactions of EtMeImAcO and glucose oligomers (5, 6, 10, and 20 monosaccharide units). These indicated that the IL is an efficient solvent, regardless of molar mass of the oligomer because of H-bonding of the acetate anion to the hydroxyl groups of the AGU. Values of |ΔH| for these interactions were EtMeImAcO > water ≈ methanol (two typical non-solvents for cellulose). The IL cation also participates in cellulose dissolution by H-bonding and solvophobic interactions [58]. The interactions of cellulose (I) crystal and water, BuMeImAcO, and BuMeImPF_6_^−^ were probed by MD, with emphasis on solvent-induced perturbation of the intra-chain (C_2_–O–H···O(H)C_6_, C_3_–O–H···O(H)C_5_) and inter-chain (C_6_–O–H···O(H)C_3_) H-bonds. Upon contact of cellulose with these three solvents, the number of H-bonds at the cellulose ensemble surface decreased, especially for BuMeImAcO. The decrease in the numbers of the intra-chain H–bonds followed the order BuMeImAcO > BuMeImPF_6_^−^ > water, indicating that the first IL has the strongest tendency to break intra-chain H-bonding, leading to cellulose dissolution [85]. The relevance of anion basicity to IL–cellulose interactions, hence biopolymer dissolution was inferred from results of density functional theory (DFT) calculations on dimethyl-CB and several anions. DFT results indicated the following order of interaction energy AcO^−^ > dimethylphosphate > BF_4_^−^ > PF_6_^−^, with the oxygen atoms of the AcO interacting simultaneously with C_2_–O***H*** and C_3_–O***H*** of the disaccharide derivative. A similar conclusion applies to the oxygen atoms of the dialkylphosphate ester. In ILs with fluorinated anions, one F^−^ (BF_4_^−^) or two F^−^ (PF_6_^−^) form weak Cel–OH·····F^−^ bonds, hence these two ILs do not dissolve cellulose [86].

**Figure 11:**
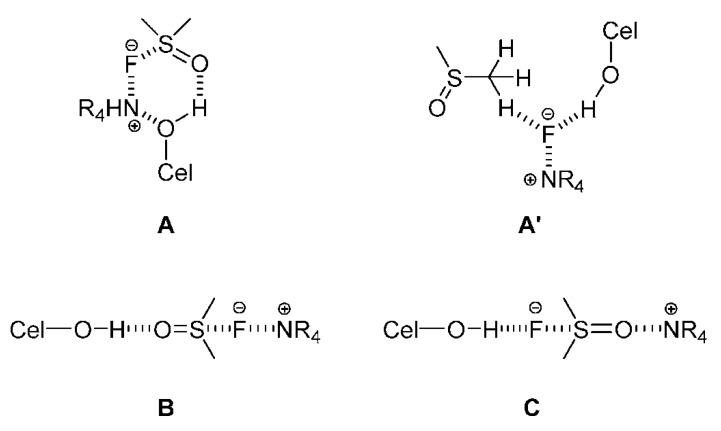
Schematic representations of cellulose/DMSO/IL structures, including the starting geometries of the aggregates (**A**–**C**), (**A’**) is an optimized geometry of (**A**), redrawn from [87], with permission from Elsevier, 2014.

We list below a few more examples to show the power of theoretical calculations to assess information that is difficult or impossible to obtain experimentally. MD indicated that electrostatics interactions between cellulose and the IL Me_2_ImCl contribute more to the total interaction energies than the van der Waals interactions. Although the interaction energy between cellulose and the IL anion is about 2.9 times that between cellulose and the cation, the role of cation is non-negligible. In contrast, the interaction energy between cellulose and water is too weak to dissolve cellulose in water [83]. For cellulose dissolved in AlMeImCl-MSs, the concentrations of both (IL) ions are larger for IL-DMSO than for (more viscous) IL-Sulf, in agreement with larger wt% dissolved cellulose, and higher reactivity (in acylation) in the former binary solvent mixture [72]. The rate constants for acetylation of cellulose in AlMeImCl-DMSO are larger than those in AlMeImCl-Sulf. The rdf curve for Cel–OH···O=S of DMSO is less structured than Cel–OH···O=S of Sulf. That is, the latter interaction is stronger because of the bidentate nature of Sulf (with two S=O bonds). This means that the reagent state of cellulose/IL-Sulf is lower energy, and more organized than that that in IL-DMSO. Hence, the reaction in IL-Sulf is expected to be associated with larger activation enthalpy and smaller |activation entropy| than in case of IL-DMSO, in agreement with the calculated activation parameters [73]. Of the structures represented below (Figure 11) for the complex Cel–OH···IL (dibenzyldimethylammonium fluoride) structure (C), and not symmetrical (A) is associated with the lowest electronic energy [87]. ILs with unsaturated heterocyclic rings (based on Im and pyridine) dissolve much more cellulose than those that carry saturated heterocyclic ring (pyrrolidine, piperidine) because of a structural factor, coupled with a dynamic effect. The first is that π-electron delocalization in the unsaturated heterocyclic ring leads to more efficient cation–cellulose interactions (H–bonding and van der Waals) and provides more “space” for acetate anions to form H-bonds with cellulose. The dynamic effect is a consequence of the larger volume of the saturated heterocyclic ring, resulting in a slow transfer of both cations and anions from bulk solvent into the cellulose ensemble; this hinders dissolution [88].

## 3. Mechanism of Regeneration of Dissolved Cellulose

The development of new, advanced, environmentally friendly solvents for cellulose dissolution and subsequent regeneration rests on a clear understanding of both mechanisms. The complex interplay between H-bonding, ionization effects, and hydrophobic interactions is crucial to control dissolution, regeneration, gelation, and related phenomena [40]. As shown above, cellulose dissolution mechanism has been investigated in detail in recent years [10,40,70,81,89,90,91,92,93,94,95,96,97,98,99,100,101]. Cellulose regeneration was less investigated due to the inherent difficulty of studying this phenomenon. Our understanding comes from the results of MD [102,103,104,105,106,107,108,109] and crystal structure analysis of, usually, regenerated fibers and membranes [110,111,112,113,114]. We give below an overview of the mechanism and results of cellulose regeneration; specific regenerated forms, e.g., fibers, films, and micro/nanospheres will not be addressed.

**Figure 12:**
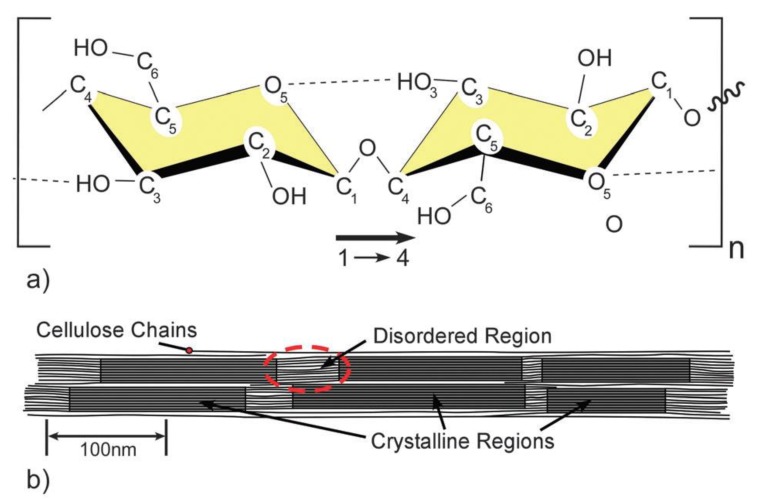
(**a**) Single cellulose chain of two anhydroglucose units (AGUs), showing the directionality of the 1–4 linkage and intra-chain H-bonding (dotted line), (**b**) idealized cellulose microfibril showing one of the suggested configurations of the crystalline and amorphous regions, adapted from [115], with permission from RCS, 2011.

**Figure 13:**
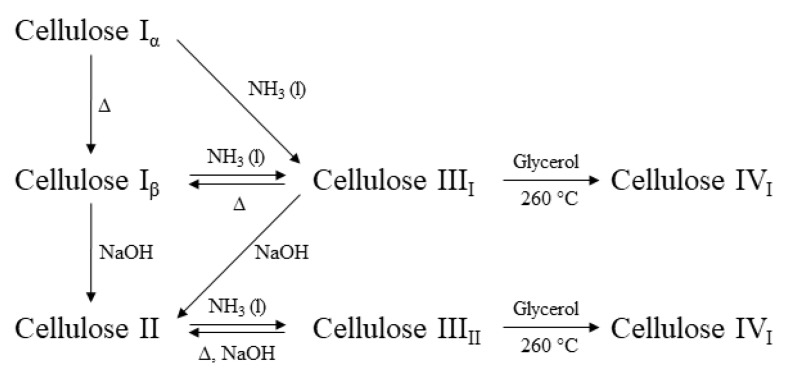
Cellulose polymorphs and their conversion, redrawn from [118], with permission from Springer, 2008.

Cellulose I is a crystalline biopolymer naturally produced by a variety of organisms (trees, plants, tunicates, algae, and bacteria), hence it is sometimes referred to as ‘‘natural’’ cellulose (Figure 12) [115]. Its structure is thermodynamically metastable and can be converted into either cellulose II or III (Figure 13). Cellulose II is the most stable structure of technical relevance and can be produced by two processes: regeneration (solubilization and recrystallization) and mercerization (aqueous alkali treatment, followed by washing) [116]. Cellulose II has a monoclinic structure, and has been used to fabricate a variety of industrial products such as cellophane (transparent films), Rayon, and Tencel (man-made cellulosic fibers). However, the intermolecular H-bonding in cellulose II is significantly more complex compared to that of cellulose I. Gold labelling of the reducing ends of cellulose II microcrystals (mercerized ramie) showed that, unlike cellulose I, the chains were packed into an antiparallel mode. Moreover, the anti-parallel chain model enables the formation of inter-chain and inter-plane H-bonds [117].

Cellulose regeneration from its solutions is based on exchange of solvent molecules with non-solvent molecules (coagulant) which initiates the reformation of cellulose [119]. It requires principally the same steps as native cellulose I crystallization: (1) formation of mini-sheets by van der Waals forces, (2) association of these sheets by H-bonding into “mini-crystals”, (3) convergence of these crystals to form the larger crystalline or amorphous arrangements [102]. As will be shown, regeneration depends on several factors, e.g., the solution itself, cellulose type employed (DP), coagulant (additives), and temperature.

**Figure 14:**
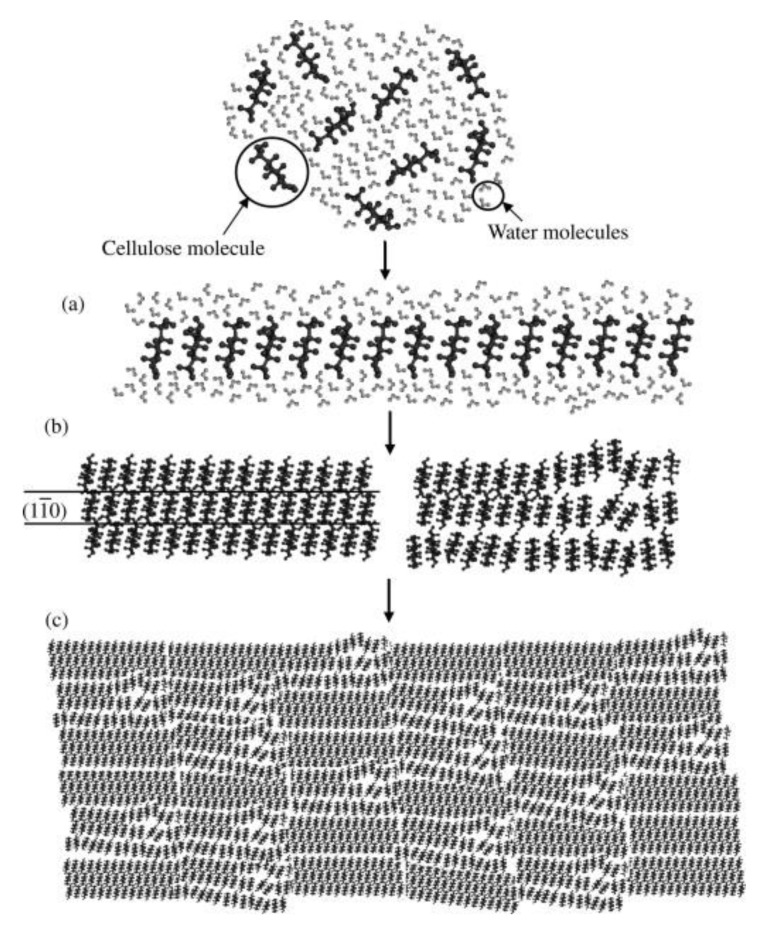
Mechanism of structure formation during the regeneration process of cellulose in aqueous solution: (**a**) formation of molecular sheets by van der Waals forces, (**b**) association of the molecular sheets by hydrogen bonds to form crystal and amorphous regions; (**c**) contact and aggregation of the structural unites to form regenerated cellulose; reprinted from [105], with permission from Elsevier, 2009.

The first experimental observation of the cellulose regeneration mechanism from LiOH/urea aqueous solution was based on time-resolved synchrotron-radiation X-ray, where the regeneration was probed by heating the solution, or adding coagulant during the measurement [112]. It was shown that the glucopyranoside rings first stack by hydrophobic interaction to form monomolecular sheets, which aligned through H-bonding to form Na-cellulose IV type crystallites (hydrate form of cellulose II with water residing between the hydrophobically stacked sheets). This regeneration process of cellulose has been hypothesized before by MD indicating that the initial structure of cellulose in solution is critical in determining its final structure [105]. Initial state therein refers to the initial cellulose solution structure composed of van der Waals-associated molecular sheets. That also includes H-bonds, molecular chain conformation, and tilt angle of the glucopyranose rings, which greatly affect the final structure of regenerated cellulose [108,120]. Figure 14 summarizes the regeneration steps:

(i) Cellulose chains aggregate side-by-side, with glucopyranose ring stacking by hydrophobic interactions and van der Waals forces, and the formation of molecular sheets of the cellulose in solution (Figure 14a);
(ii) Sheet association by H-bonds with progressively growing stacks, to form thin planar crystals incorporating amorphous chains as well (Figure 14b, right). Note that the amorphous regions are defined as molecular sheets with non-uniform distances between each other [121,122];
(iii) Diffusion controlled aggregation of bigger three-dimensional clusters of the randomly dispersed structures in solution to form a mixture of crystal and amorphous regions (Figure 14c).

**Figure 15:**
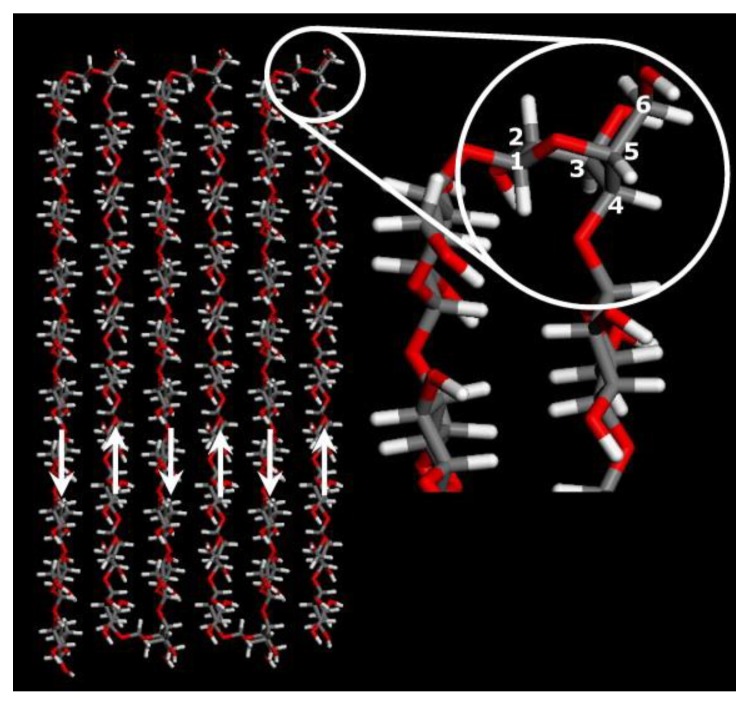
The folded-chain structure linked by the boat ring conformer to allow the “hairpin” turn (magnification inset). The white arrows show the anti-parallel orientation of the cellulose chains, grey–carbon atoms, red–oxygen atoms, white–hydrogen atoms, numbers 1–6 refer to the conventional numeration of carbon atoms of the anhydroglucose unit (AGU);reprinted from [108], with permission from Elsevier, 2013.

Further MD suggest the possibility of forming folded cellulose chains during the regeneration process [108]. While it is impossible for cellulose I chains to fold due to their parallel orientation, the anti-parallel orientation in cellulose II allows the crystal to be ordered in a folded-chain packing by forming a “hairpin” (see amplification of Figure 15). Based on the similarity between folded and extended chains in the cellulose II crystal regarding their energy and lattice parameters, it is likely for both to coexist in the regenerated biopolymer. A single folded-chain molecular sheet might as well be an initial structure during crystallization towards larger crystals. In conclusion, the initial regeneration step does not start with the reformation of broken H-bonds but rather with the hydrophobic stacking of the glucopyranose rings. These aforementioned findings are important for a better understanding of cellulose dissolution too, albeit in inverse order.

In order to shed light on crystalline order and crystal structure of cellulose films, MD (gas- or water) were conducted [106]. Optimized interface structures of different cellulose allomorphs were constructed from crystallographic data. Cellulose crystal structural properties such as unit cell parameters, dihedral conformation distributions, density profiles, and H-bonding were calculated for the biopolymer bulk and surface. A cellulose H-bonded network is reconstructed within the first few layers. Water molecules near the cellulose ensemble surface adsorb preferentially around the terminal hydroxyl groups whereas those H-bonded to the glucopyranose ring are depleted. Moreover, water molecules showed reduced mobility close to the cellulose surface.

To show intra and intermolecular hydrogen bonding, a cellulose II film was investigated by polarized FTIR [123]. The bands at 3491 and 3447 cm^−1^ were assigned to intramolecular H-bonds because these bonds preferentially form along the direction of the long axis of *β*-1,4-glucan chains. The remaining bands at 3353, 3276, and 3162 cm^−1^ were assigned to the intermolecular H-bonds between *β*-1,4-glucan chains. Furthermore, there are two types of OH bands related to intramolecular hydrogen bonds and three types of OH bands related to intermolecular hydrogen bonds in cellulose II.

Alkali-induced cellulose mercerization is an important industrial operation and is used as pre-treatment in the synthesis of many cellulose derivatives [124]. The influence of different solvents, additives, temperatures, times, external pressures, and the concentration of alkali on the transformation from cellulose I to cellulose II was studied by X-ray diffraction. Alcohols, e.g., 2-propanol, and its mixture with ethanol are the solvents of choice for slurry mercerization (with total alcohol concentration between 80 and 90 wt%); aqueous 2-propanol is superior to aqueous ethanol because of the limited solubility of NaOH in the latter binary solvent mixture. Alkali induced cellulose I ⟶ cellulose II structure transformation (NaOH and NaOH/urea) was studied by wide-angle X-ray diffraction analysis. [113]. As expected, water alone does not induce structure transformation. Extent of the latter depended on the concentration of NaOH; slight at 5 wt% NaOH, intensive at 15 wt% NaOH. This sudden transformation at 15 wt% NaOH indicates that a defined swelling and spacing is required before the actual cellulose I ⟶ cellulose II transformation takes place. Therefore, mercerization is best described in terms of an intermingling of the cellulose chains from neighboring swollen micro-fibrils of opposite polarity [125]. In the presence of 5 wt% urea and 15 wt% NaOH the magnitude of the transformation is reduced largely. Thus, urea inhibits rearrangement of the cellulose polymorphs most likely by instant formation of new, more stable H-bonds that were previously split by NaOH [113].

The coagulation during cellulose regeneration processes is a crucial step in determining the final properties of the regenerated cellulose products. This is illustrated by the fact that the mechanical properties of cellulose films regenerated from different cellulose sources in ILs (1-ethyl-3-methylimidazolium acetate) are greatly improved because of the synergistic effect of crystallinity and increasing DP value [114]. The effect of the coagulant on cellulose membrane formation from NaOH/urea solutions was studied. The solutions were coagulated in either distilled water, 5 wt% H_2_SO_4_, or 5 wt% (NH_4_)_2_SO_4_ [126]. Membranes obtained from distilled water had the highest tensile strength and crystallinity index but showed the lowest value for elongation at break. The opposite trend was observed for membranes obtained by coagulation with 5 wt% (NH_4_)_2_SO_4_ solution. The higher tensile strength was attributed to the compact structure of regenerated membrane due to stronger inter- and intra-molecular hydrogen bonding. Post-treatment of the regenerated cellulose films with different solvents, e.g., hexane, liquid ammonia, or hot glycerol can somewhat control the hydrophilicity/hydrophobicity of their surfaces, because of the inherent structural anisotropy of the AGU [111]. E.g., liquid ammonia and hot glycerol post-treatment transformed cellulose II into cellulose III and cellulose IV, respectively. These findings were supported by contact angle measurements as well.

The relevance of investigating regeneration of different physical forms, including fibers and nanoparticles is shown by the fact that fibers obtained by regeneration of cellulose dissolved in NaOH/urea (or LiOH/urea) lack stability and good mechanical properties (tensile strength and elongation). This limits practical application of the obtained wet-spun filaments in the industry [127,128,129]. This limitation can be mitigated by using additives. E.g., addition of small concentrations of phytic acid seemingly promotes a rapid diffusion from the skin to the core of the cellulose filament, resulting in a relatively compact structure [130]. The overall mechanical properties of the fibers spun are comparable to commercial viscose.

## 4. Conclusions

The impetus for the increased interest in developing efficient, environmentally acceptable, recyclable cellulose solvents is the expected cellulosic-fiber gap. Organic electrolytes, pure and as solutions in dipolar aprotic solvents dominated the research as solvents for the physical dissolution of cellulose. Structural flexibility is probably the most important favorable characteristic of these ionic compounds. This means that the macroscopic (e.g., viscosity and surface tension) and microscopic properties (e.g., Lewis acidity and basicity) of the solvents can be fine-tuned according to our need. The most important classes are ionic liquids, especially those based on imidazole and quaternary ammonium ions. Some salts of super-bases are gaining importance because of their ease of synthesis and the recovery (by removal of the volatile carboxylic acid).

The choice of a solvent that dissolves cellulose physically rests on understanding the biopolymer dissolution mechanism. This is a cooperative process: the (basic) anion interacts with the hydroxyl groups of the AGU, the organic cation condenses around the negatively charged cellulose-anion complex. Both effects lead to disruption of the intra- and intermolecular H-bonds and van de Waals interactions, leading to cellulose dissolution.

A usual approach is to synthesize, and then assess the efficiency of a series of ILs, or their binary mixtures with MSs, then to rationalize the order observed in terms of the macroscopic, and microscopic properties of the solvent, a consequence of the molecular structure of the IL. We discussed the determinations of these properties as well as how we rationalize the results in terms of solute (cellulose)–solvent interactions. Solvatochromic parameters are determined by use of solvatochromic probes. These are compounds sensitive to a particular microscopic property that is relevant to cellulose dissolution, e.g., Lewis acidity and Lewis basicity. The picture that emerges based on these parameters is that high SB (or SB-SA), and SD/SP are of paramount importance for cellulose dissolution. Additionally, low viscosity of the resulting solution, shear thinning, and absence of steric crowding or structural rigidity favor cellulose dissolution.

Other experimental techniques and theoretical calculations were used to gain insight into the mechanism of cellulose–solvent interactions. FTIR and **^1^**H, **^13^**C NMR is particularly suitable because (ν**_OH_**) and the chemical shifts and relaxation times of CB and the IL provide direct evidence for IL···AGU H-bonding. Finally, we showed how theoretical calculations corroborate the experimental results by providing information about partial charges, average distance (e.g., between the IL anion and the OH group of the AGU), and the average composition of the solvation layer of the cellulose assembly. Other techniques that contributed to our understanding of cellulose physical dissolution in these solvents include rheology and conductivity.

Cellulose regeneration studies are as important as dissolution studies. Therefore, MD simulations, crystal structure analysis, and IR of regenerated fibers and membranes have been carried out. The results suggest three steps for the regeneration mechanism: (i) glucopyranose rings of the cellulose chains stack by hydrophobic interactions and van der Waals forces to form molecular sheets; (ii) the formed sheets associate by hydrogen bonding to form thin planar crystals; (iii) aggregation into bigger three-dimensional clusters with crystalline and amorphous regions. These findings are essential in order to control cellulose regeneration in dependence of the desired product properties.

## Abbreviations

AcO	Acetate group
AGU	Anhydroglucose unit
Bu	1-(*n*-Butyl) group
BuMeImX	1-(n-Butyl)-3-methylimidazolium electrolyte, X = anion
BuO	Butanoate
CB	Cellobiose
CT	Charge transfer
DBN	1,5-Diazabicyclo [4.3.0]non-5-ene
DBU	1,8-Diazabicyclo [5.4.0]undec-7-ene
DMAc	*N,N*-Dimethylacetamide
DMF	*N,N*-Dimethylformamide
DMI	1,3-Dimethyl-2-imidazolidinone
DMSO	Dimethylsulfoxide
DP	Average degree of polymerization
*E_T_*(probe)	Solvent empirical polarity in kcal·mol**^−1^**, calculated from solvatochromic data
*E_T_*(RB)	Solvent empirical polarity scale based on the probe 2,6-diphenyl-4-(2,4,6-triphenylpyridinium-1-yl)phenolate
*E_T_*(WB)	Solvent empirical polarity scale based on the probe 2,6-dichloro-4-(2,4,6-triphenylpyridinium-1-yl)phenolate
EtMeImAcO	1-Ethyl-3-methylimidazolium acetate
EtMeIm(EtO)_2_PO_2_	1-Ethyl-3-methylimidazolium diethylphosphate
IL	Ionic liquid
Im	Imidazole
MCC	Microcrystalline cellulose
MD	Molecular dynamics
Me	Methyl group
MS	Molecular solvent
N(CN)_2_	Dicyanamide anion
PrO	Propionate group
PrOMeImAcO	1-(2-Methoxyethyl)-3-methylimidazolium acetate
QAE	Quaternary ammonium electrolytes, a sub-class of ionic liquids.
S	Solvent
SA	Solvent Lewis acidity; calculated from solvatochromic data
SB	Solvent Lewis Basicity; calculated from solvatochromic data
SD	Solvent dipolarity; calculated from solvatochromic data
SP	Solvent polarizability; calculated from solvatochromic data
Sulf	Sulfolane
TBAF·xH_2_O	Tetra(*n*-butyl)ammonium fluoride hydrate
Tf_2_N	Bistriflimide anion
TMG	Tetramethylguanidine
V	Volume
χ	Mole fraction
λ	Wavelength
